# *ZmCaM2-1*, a Calmodulin Gene, Negatively Regulates Drought Tolerance in Transgenic *Arabidopsis* Through the ABA-Independent Pathway

**DOI:** 10.3390/ijms26052156

**Published:** 2025-02-27

**Authors:** Zhiqiang Wu, Meiyi Liu, Hanqiao Wang, Mingrui Li, Xiaoyue Liu, Zhenyuan Zang, Liangyu Jiang

**Affiliations:** College of Agriculture, Jilin Agricultural University, Changchun 130118, China; 17396774079@163.com (Z.W.); l2932144311@163.com (M.L.); wanghanqiao02@163.com (H.W.); mqmqlee@163.com (M.L.); iuiaoue@163.com (X.L.)

**Keywords:** maize, drought stress, *ZmCaM2-1*, abscisic acid, reactive oxygen species

## Abstract

Calmodulin (CaM) family members play crucial roles in the response to various abiotic stresses. However, the functions of CaMs in the response to drought stress in maize are unclear. In this study, a CaM gene, *ZmCaM2-1*, was isolated from the maize (*Zea mays L.*) inbred line B73. The coding sequence (CDS) of *ZmCaM2-1* was 450 bp with a protein of 149 aa which contains four EF-hand motifs. The ZmCaM2-1 protein was located in the cell nucleus and membrane, and is able to bind to Ca^2+^. *ZmCaM2-1* was strongly induced by drought, NaCl, and low-temperature treatments, except for abscisic acid (ABA) treatment. Overexpression of *ZmCaM2-1* in *Arabidopsis* was found to decrease the drought tolerance with lower antioxidant enzyme activity and greater reactive oxygen species (ROS) production. Moreover, there was no significant difference in the phenotype and ABA-related gene expression levels between *ZmCaM2-1*-overexpressing *Arabidopsis* and the wild type (WT) under ABA treatment. These results indicate that *ZmCaM2-1* negatively regulates the tolerance of *Arabidopsis* to drought stress through the ABA-independent pathway.

## 1. Introduction

Maize is a major food, economic, and animal feed crop which plays a vital role in ensuring food security worldwide. However, its growth and development are often threatened by various abiotic stresses, including drought, salinity, heat, and cold [1,2]. Of these, drought stress is the most important limiting factor, leading to severe production losses ranging from 20% to 40% [3]. Therefore, exploring drought-tolerance-related genes or studying the transcription factors that trigger these genes’ up- or down-regulation [4] and developing drought-tolerant varieties are of great importance.

The calcium ion (Ca^2+^), as a significant second messenger, plays a key role in the response to various abiotic and biotic stresses [5]. When exposed to external stress, the concentration of free Ca^2+^ in plants changes. Subsequently, plants decode and transmit Ca^2+^ messages through Ca^2+^ sensors [6]. To date, several Ca^2+^ sensors have been identified in plants, including CaM/CML (calmodulin and calmodulin-like protein), CBL (calcineurin B-like protein), and CDPK (calcium-dependent protein kinase) [7]. Unlike the CDPK and CBL proteins, CaM/CML proteins only possess EF-hand domains, which have been found to function as a Ca^2+^ binding site [8,9]. CaM and CML proteins contain four EF-hand domains and between one and six EF-hand domains, respectively [10].

CaM and CML have been shown to be involved in the response to drought stress [11]. Overexpression of *OsDSR-1* can enhance the tolerance of rice to drought stress [12]. *CmCML13*-overexpressing *Arabidopsis* plants exhibit stronger drought stress tolerance [13]. *AtCML37* knock-out *Arabidopsis* shows reduced drought stress tolerance [14]. Overexpression of *MtCaMP1* significantly enhances the tolerance of *Arabidopsis* to drought stress [15]. Overexpression of *VaCML92* leads to increased drought tolerance in *Arabidops*is [16]. *AtCML9* negatively regulates the drought tolerance of *Arabidopsis* [17]. These findings demonstrate that the *CaM* and *CML* genes play important roles in regulating drought stress responses.

Several studies have shown that Ca^2+^ is involved in responses to drought stress though the abscisic acid (ABA)-dependent and ABA-independent signaling pathways, and some Ca^2+^ sensors have been found to act as core transductors [18]. *CML37*, *CML38*, and *CML39* are induced by drought and ABA treatments [19]. *OsMSR2* has been shown to be involved in drought stress tolerance through the ABA-dependent signaling pathway [20]. *AtCML42* loss-of-function mutants show enhanced tolerance to drought stress through the ABA-dependent signaling pathway [21]. However, *OsCML4* was found to increase the tolerance of plants to drought stress through the ABA-independent signaling pathway [22]. These findings suggest that *CaM* and *CML* genes can regulate drought stress tolerance through the ABA-dependent and ABA-independent signaling pathways.

An increasing number of studies have shown that the *CaM* and *CML* genes are involved in drought stress responses through the reactive oxygen species (ROS) signaling pathway. Overexpression of *OsCML4* can confer enhanced drought tolerance through clearing ROS accumulation [22]. Overexpression of *MsCML46* can improve the tolerance of tobacco to freezing, drought, and salt stresses by decreasing the production of ROS [23]. Overexpression of *ShCML44* can increase the tolerance of tobacco to cold, drought, and salt stress by reducing ROS accumulation [24]. *HvCRK2* and *HvCRK4* were found to interact with *HvCML32* and negatively regulate drought tolerance by enhancing ROS accumulation [25]. These studies demonstrated that the *CaM* and *CML* genes can modulate drought stress tolerance through affecting ROS accumulation.

The *CaM* and *CML* genes play an important role in the response to drought stress. However, the functions of the *CaM* and *CML* genes in maize remain to be comprehensively elucidated. Previously, we identified the *CaM* and *CML* genes in maize, and used a transcriptome sequencing database to find that a *CaM* gene expression was significantly up-regulated under drought stress in maize, suggesting that it may be involved in drought stress tolerance [26,27]. In this study, the *CaM* gene *ZmCaM2-1* was cloned from the maize inbred line B73. A function analysis of *ZmCaM2-1* was carried out through subcellular localization and Ca^2+^ binding analysis. We revealed the function of *ZmCaM2-1* under drought stress based on *ZmCaM2-1*-overexpressing transgenic *Arabidopsis*. The findings highlight the potential role of *ZmCaM2-1* in the response to drought stress and provide an important foundation for breeding drought-tolerant maize varieties.

## 2. Results

### 2.1. Gene Cloning and Sequence Analysis of ZmCaM2-1

**Figure 1:**
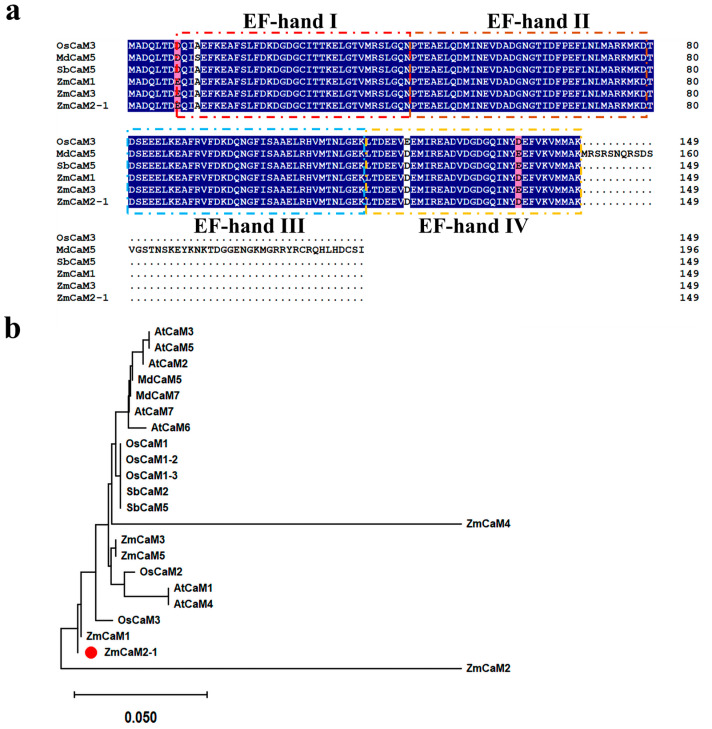
Sequence alignment and phylogenetic analysis of ZmCaM2-1. (**a**) Alignment of amino acid sequences of ZmCaM2-1. The EF-hand domains are indicated by boxes. (**b**) Phylogenetic analysis of ZmCaM2-1 protein. The accession numbers are as follows: AtCaM1 (AT5G37780.1), AtCaM2 (AT2G41110.1), AtCaM3 (AT3G56800.1), AtCaM4 (AT1G66410.1), AtCaM5 (AT2G27030.1), AtCaM6 (AT5G21274.1), AtCaM7 (AT3G43810.1), OsCaM1 (LOC_Os03g20370.1), OsCaM1-2 (LOC_Os07g48780.1), OsCaM1-3 (LOC_Os01g16240.1), OsCaM2 (LOC_Os05g41210.1), OsCaM3 (LOC_Os01g17190.1), MdCaM5 (MD03G1163500), MdCaM7 (MD12G1111300), SbCaM2 (Sobic.001G390300.1), SbCaM5 (Sobic.003G125650.1), ZmCaM1 (Zm00001d028948), ZmCaM3 (Zm00001d038543), ZmCaM4 (Zm00001d038545), ZmCaM5 (Zm00001d022546). The different background colors represent the similar degree of amino acid sequences. Blue: the similar degree of amino acid sequences is equal to 100%. Pink: the similar degree of amino acid sequences is less than 100% and greater than or equal 75%. White: the similar degree of amino acid sequences is greater than 50%. The red dot indicates the *ZmCaM2-1* gene (Zm00001d040323).

The coding sequence (CDS) of *ZmCaM2-1* (Zm00001d040323) was cloned from leaves of the inbred maize B73. The CDS length of *ZmCaM2-1* was 450 bp and encoded a protein of 149 amino acids with a predicted mass of 19.8 kDa and a theoretical PI of 10.15. Multiple sequence alignment of ZmCaM2-1 with its orthologs found that ZmCaM2-1 possesses four conserved EF-hand domains (Figure 1a). To investigate the evolutionary relationships of ZmCaM2-1, a phylogenetic tree was constructed using MEGA11. The results showed that the ZmCaM2-1 amino acid had the highest degree of homology with *Oryza sativa* OsCaM3 (99%), followed by *Sorghum bicolor* (L.) *Moench* SbCaM2 (99%) and *Arabidopsis thaliana* AtCaM7 (98%) (Figure 1b).

### 2.2. Expression Profiling of ZmCaM2-1 Under Various Treatments

**Figure 2:**
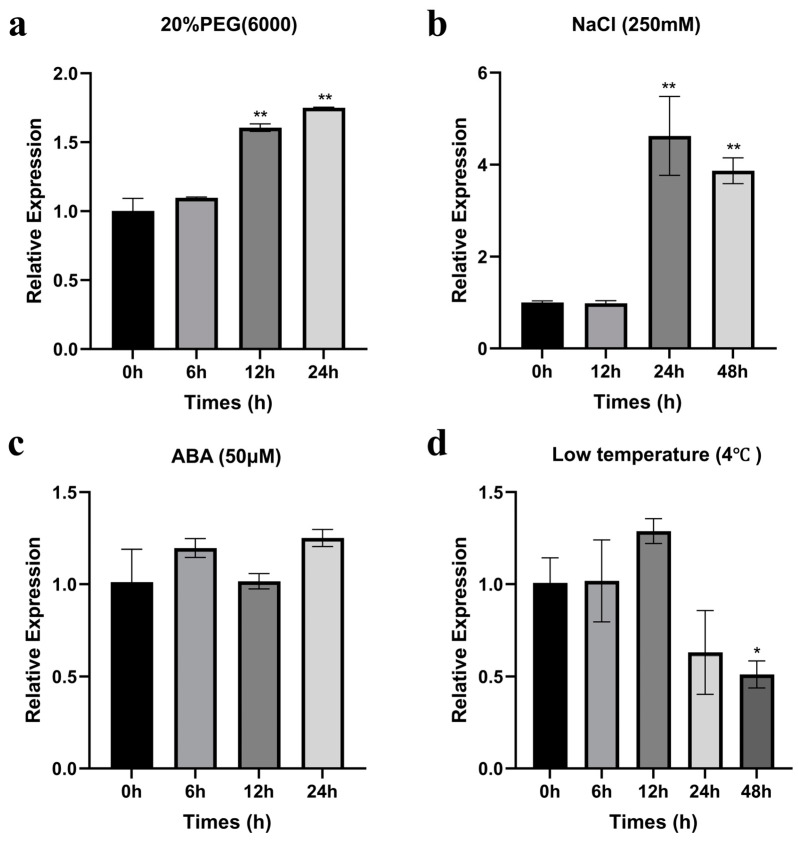
The relative expression levels of *ZmCaM2-1* under various treatments: (**a**) 20% PEG6000 treatment, (**b**) 250 mM NaCl treatment, (**c**) 50 μM ABA treatment, and (**d**) low-temperature treatment (4 °C). The relative expression levels were analyzed using the 2^−ΔΔCT^ method. The significance analysis compared with 0 h was performed using one-way ANOVA (* *p* < 0.05, ** *p* < 0.01). Bars indicate standard error of the mean. The experiment was performed using three biological replicates.

To explore the underlying function of *ZmCaM2-1*, we further assessed the transcription levels of *ZmCaM2-1* under PEG, NaCl, ABA, and low-temperature treatments in maize by using Quantitative Real-Time PCR (qRT-PCR). After 20% PEG6000 treatment, the relative expression level of *ZmCaM2-1* was significantly up-regulated from 12 h to 24 h and reached its highest level at 24 h (1.7 fold) (Figure 2a). After 250 mM NaCl treatment, the relative expression level of *ZmCaM2-1* peaked at 24 h, and was 4.6 times higher than that at 0 h (Figure 2b). The relative expression level of *ZmCaM2-1* showed no significant change after exogenous 50 μM ABA treatment (Figure 2c). After low-temperature treatment, the relative expression level of *ZmCaM2-1* increased from 6 h to 12 h, and then gradually decreased from 24 h to 48 h (Figure 2d). Taken together, these results indicate that *ZmCaM2-1* expression is significantly induced by drought, salt, and low-temperature treatments, except when under ABA treatment.

### 2.3. The ZmCaM2-1 Protein Is Located in the Cell Nucleus and Membrane

**Figure 3:**
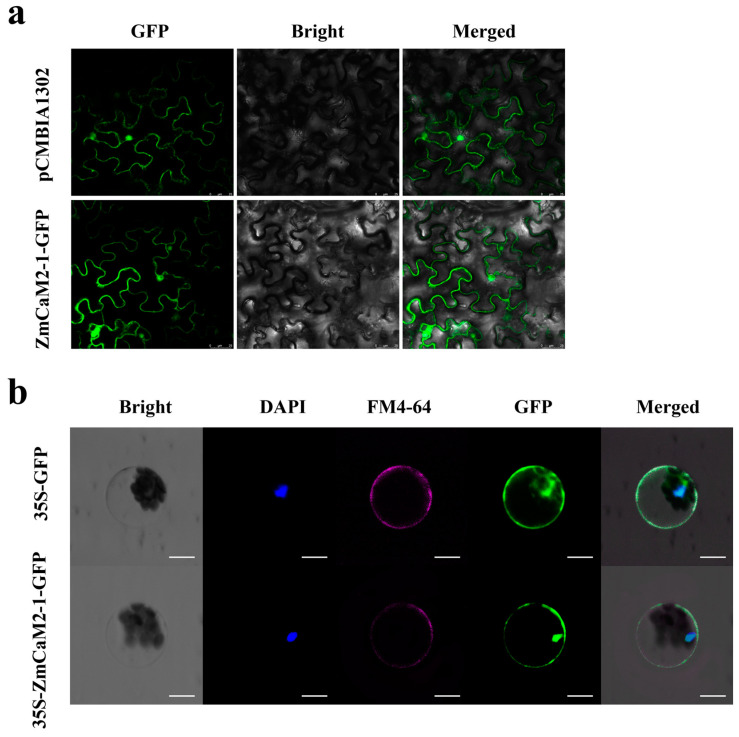
Subcellular location of ZmCaM2-1-GFP in the plant cell. (**a**) ZmCaM2-1-GFP was expressed in *Nicotiana benthamiana* leaf epidermal cells. GFP alone was used as a control. Bar = 25 μm. (**b**) ZmCaM2-1-GFP was expressed in *Arabidopsis* protoplasts. GFP alone was used as a control. Nuclear (staining with DAPI) and the membrane (staining with FM4-64) fluorescent signals are labeled blue and red, respectively. Bar = 10 μm.

To determine the subcellular localization of the ZmCaM2-1 protein, a ZmCaM2-1- green fluorescent protein (GFP) fusion protein was constructed and transformed into the leaves of *Nicotiana benthamiana* by the *Agrobacterium*-mediated method. The pCAMBIA1302-GFP vector was used as a control. As shown in Figure 3a, ZmCaM2-1-GFP was observed in the cell nucleus and membrane. The control-GFP showed similar results. To further verify the location of the ZmCaM2-1 protein, a 35S:: ZmCaM2-1-GFP vector was constructed and transiently expressed into *Arabidopsis* protoplasts. As shown in Figure 3b, the control was observed throughout the whole cell, while 35S:: ZmCaM2-1-GFP was observed only in the cell membrane and nucleus. These results demonstrate that the ZmCaM2-1 protein is located in the cell membrane and nucleus.

### 2.4. ZmCaM2-1 Is Able to Bind to Ca^2+^

**Figure 4:**
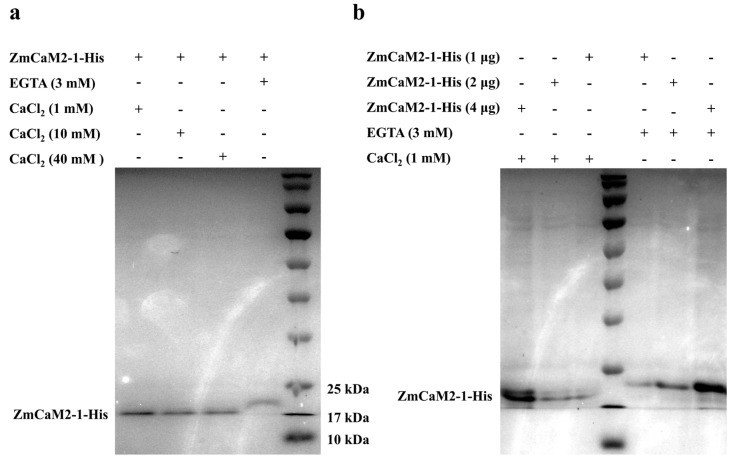
The SDS-PAGE mobility shift assay demonstrates that ZmCaM2-1 can bind to Ca^2+^. (**a**) Different concentrations of CaCl_2_ (1 mM, 10 mM, and 40 mM) or 3 mM EGTA were added to the purified ZmCaM2-1-His protein. (**b**) Amounts of 1 mM CaCl_2_ or 3 mM EGTA were added to the different concentrations of ZmCaM2-1-His protein (1 μg, 2 μg, 4 μg). The (+) and (−) indicate the presence and absence, respectively.

The ZmCaM2-1 protein has four conserved EF-hand domains, indicating that it can probably bind to Ca^2+^. To investigate whether ZmCaM2-1 can bind to Ca^2+^, a 15% SDS-PAGE mobility shift analysis was performed in the presence of either (1 mM, 10 mM, and 40 mM) CaCl_2_ or 3 mM ethylene glycol tetraacetic acid (EGTA). Conserved CaM typically shows a more rapid electrophoretic migration in the presence of Ca^2+^ than in the presence of EGTA [28]. As shown in Figure 4a, the migration rate of the recombinant protein ZmCaM2-1-His was slower in the presence of an EGTA chelator than in the presence of Ca^2+^. When different contents of ZmCaM2-1-His (1 μg, 2 μg, and 4 μg) were added with 1 mM CaCl_2_ or 3 mM EGTA, the ZmCaM2-1-His migrated faster in the presence of Ca^2+^ than in the presence of EGTA (Figure 4b). These results demonstrate that ZmCaM2-1 is able to bind to Ca^2+^.

### 2.5. Overexpression of ZmCaM2-1 Decreases the Tolerance of Arabidopsis to Drought Stress

**Figure 5:**
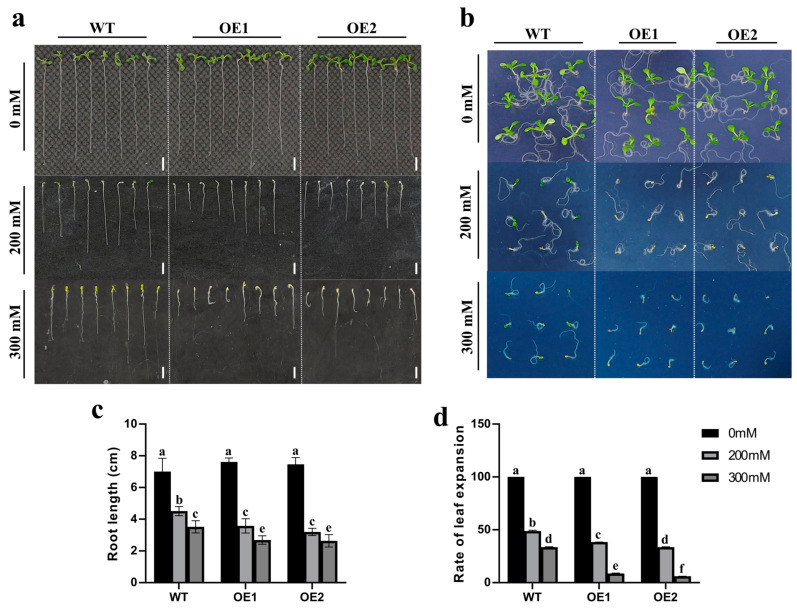
*ZmCaM2-1*-overexpressing lines displayed inhibited root length and leaf expansion rate under 200 mM and 300 mM mannitol treatments, respectively. (**a**) Phenotype of *ZmCaM2-1*-overexpressing lines (OE1 and OE2) and wild-type (WT) *Arabidopsis* (Columbia) seedlings’ root length under normal growth conditions (1/2 MS), and under 200 mM or 300 mM mannitol treatment. Scale bars = 1 cm. (**b**) Phenotype of leaf expansion rate in OE1, OE2, and WT under normal growth conditions, and under 200 mM or 300 mM mannitol treatment, respectively. (**c**) Root lengths of *ZmCaM2-1*-overexpressing lines and WT were analyzed under 200 mM or 300 mM mannitol treatment. (**d**) Leaf expansion rates of *ZmCaM2-1*-overexpressing lines and WT were analyzed under 200 mM or 300 mM mannitol treatment. The significance analysis compared with WT was performed using two-way ANOVA (different lowercase letters indicate a difference at the 0.01 level *p* < 0.01). Bars indicate standard error of the mean. The experiment was performed using three biological replicates.

To explore the role of *ZmCaM2-1* in the response to drought stress, *ZmCaM2-1* was overexpressed in wild-type (WT) *Arabidopsis* (Columbia). The T3-generation *ZmCaM2-1*-overexpressing lines (OE1 and OE2) with the highest expression (Figure S1) were selected for further analysis. Under normal conditions, there are no significant differences in the length of the roots between the WT and the *ZmCaM2-1*-overexpressing lines. However, under 200 mM mannitol or 300 mM mannitol treatment, root growth was more inhibited in OE1 and OE2 than in the WT. Under 200 mM mannitol treatment, the root lengths of OE1 (3.6 cm) and OE2 (3.2 cm) were significantly shorter than those of the WT (4.5 cm). Under 300 mM mannitol treatment, the inhibition effect was more obvious, and the root length of the WT was 3.5 cm, while that of OE1 and OE2 was 2.7 cm and 2.6 cm, respectively (Figure 5a,c). The leaf expansion rate was analyzed under 200 mM mannitol or 300 mM mannitol treatment. There were no significant differences in the leaf expansion rate between the WT (100%) and the *ZmCaM2-1*-overexpressing lines (100%) under normal conditions. However, the leaf expansion rate was significantly lower in the *ZmCaM2-1*-overexpressing lines than in the WT. Under 200 mM mannitol treatment, the leaf expansion rate in the WT was 49.8%, but was 38.2% and 33.8% in the OE1 and OE2, respectively. Under 300 mM mannitol treatment, the leaf expansion rate in OE1 (8.7%) and OE2 (5.9%) was lower than in the WT (33.7%) (Figure 5b,d). These results indicate that overexpression of *ZmCaM2-1* negatively regulates the tolerance of *Arabidopsis* to mannitol treatment.

**Figure 6:**
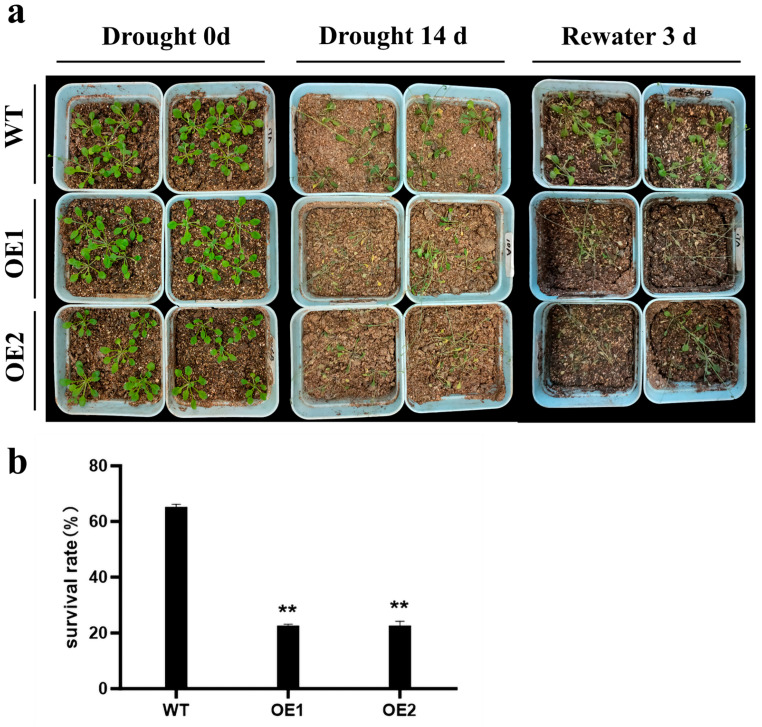
*ZmCaM2-1*-overexpressing lines show decreased tolerance to drought stress. (**a**) The three-week-old plants were subjected to drought treatment by withholding water for 14 d, and then re-watering for 3 d. (**b**) The survival rate was analyzed by re-watering for 3 d. The significance analysis compared with WT was performed using one-way ANOVA (** *p* < 0.01). Bars indicate standard error of the mean. The experiment was repeated three times with similar results.

To further verify whether overexpression of *ZmCaM2-1* decreased the tolerance of *Arabidopsis* to drought stress, 3-week-old OE1, OE2, and WT seedlings were subjected to drought treatment by withholding water for 14 days (d), followed by re-watering for 3 d. As shown in Figure 6a, the *ZmCaM2-1*-overexpressing lines (OE1 and OE2) exhibited a more severely wilted phenotype than the WT. After watering was resumed, the growth of the *ZmCaM2-1*-overexpressing lines was weaker than in the WT. The survival rate of the WT was 65.3%, while that of OE1 and OE2 was only 22.67% and 22.69%, respectively (Figure 6b). Taken together, our results demonstrate that overexpression of *ZmCaM2-1* negatively regulates the drought tolerance of *Arabidopsis*.

### 2.6. Overexpression of ZmCaM2-1 Decreases Drougth Stress Tolerance Through Increasing ROS Accumulation

**Figure 7:**
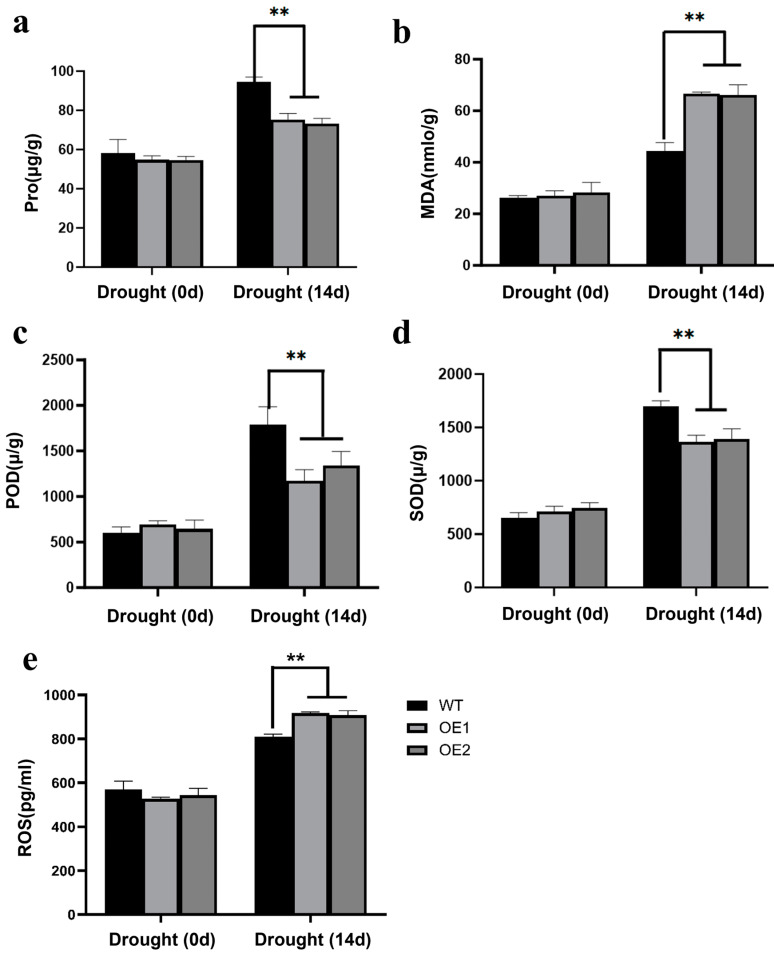
The physiological indicators were measured in *ZmCaM2-1*-overexpressing lines after drought treatment for 14 d: (**a**) Pro content, (**b**) MDA content, (**c**) POD activity, (**d**) SOD activity, (**e**) ROS content. The significance analysis compared with WT was performed using two-way ANOVA (** *p* < 0.01). Bars indicate standard error of the mean. The experiment was performed using three biological replicates.

To explore the function of *ZmCaM2-1* in the response to drought stress, we measured various physiological indicators of the *ZmCaM2-1*-overexpressing lines and the WT after drought treatment for 14 d, including proline (Pro) content, malondialdehyde (MDA) content, the activity of superoxide dismutase (SOD) and peroxidase (POD), and ROS content. Under normal conditions, there were no significant differences in SOD activity, POD activity, Pro content, MDA content, or ROS content between the WT and the *ZmCaM2-1*-overexpressing lines (OE1, OE2). However, after drought stress treatment for 14 d, the Pro content and the POD and SOD activity were significantly lower in OE1 and OE2 than in the WT (Figure 7a,c,d). The MDA content was significantly higher in OE1 and OE2 than in the WT (Figure 7b). Meanwhile, the ROS content was significantly greater in OE1 and OE2 than in the WT under drought treatment (Figure 7e). These results demonstrate that overexpression of *ZmCaM2-1* decreases the tolerance of *Arabidopsis* to drought stress by increasing the ROS and MDA content, and decreasing the Pro content and the POD and SOD activity.

### 2.7. The Transgenic Arabidopsis Shows Normal Sensitivity to ABA Thanks to the Overexpression of the ZmCaM2-1

**Figure 8:**
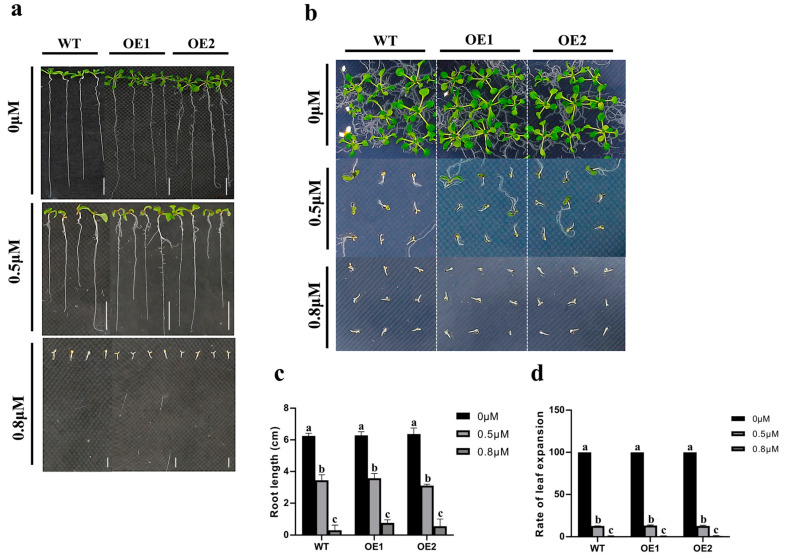
Root length and leaf expansion rate showed no significant change in *ZmCaM2-1*-overexpressing lines under 0.5 μM and 0.8 μM ABA treatments, respectively. (**a**) Phenotype of root length in *ZmCaM2-1*-overexpressing lines (OE1 and OE2) and WT under normal 0 μM, 0.5 μM, or 0.8 μM ABA treatments. Scale bars = 1 cm. (**b**) Phenotype of leaf expansion rate in OE1, OE2, and WT under 0 μM, 0.5 μM, or 0.8 μM ABA treatments, respectively. (**c**) Root length of *ZmCaM2-1*-overexpressing lines under ABA treatment. (**d**) Leaf expansion rate of *ZmCaM2-1*-overexpressing lines under ABA treatment. The significance analysis compared with WT was performed using two-way ANOVA (different lowercase letters indicate a difference at the 0.01 level *p* < 0.01). Bars indicate standard error of the mean. The experiment was performed using three biological replicates.

To determine whether *ZmCaM2-1* was associated with the ABA signaling pathway, the root length and leaf expansion rate of the WT and the *ZmCaM2-1*-overexpressing lines (OE1 and OE2) were measured under 0 μM, 0.5 μM, and 0.8 μM ABA treatment, respectively. As shown in Figure 8, there were no significant differences in the root length and leaf expansion rate between the WT and the *ZmCaM2-1*-overexpressing lines under 0 μM ABA treatment. The root length and leaf expansion rate of the *ZmCaM2-1*-overexpressing lines and the WT also showed no significant differences under 0.5 μM or 0.8 μM ABA treatment, respectively. The *ZmCaM2-1*-overexpressing lines displayed normal sensitivity to ABA, suggesting that *ZmCaM2-1* is independent of the ABA signaling pathway under drought stress.

### 2.8. Overexpression of ZmCaM2-1 Reduces the Expression of Drought-Related Genes but Has No Effect on ABA-Related Genes

**Figure 9:**
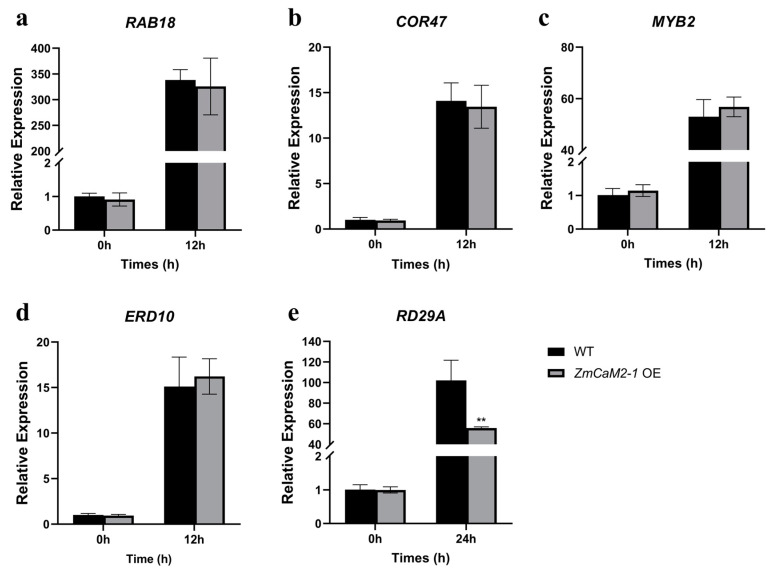
The relative expression levels of the drought-stress-responsive and ABA-responsive genes were analyzed in WT and *ZmCaM2-1*-overexpressing lines, including (**a**) *RAB18* (At5g66400), (**b**) *COR47* (At1g20440), (**c**) *MYB2* (At2g47190), (**d**) *ERD10* (At1g20450), (**e**) *RD29A* (At5g52310). The expression of ABA-responsive genes (**a**–**d**) was analyzed in WT and *ZmCaM2-1*-overexpressing lines by exposure to 0.8 µM ABA for 12 h. The expression of the drought-stress-responsive gene (**e**) was analyzed by drought stress treatment for 24 h. The relative expression levels were analyzed using the 2^−ΔΔCT^ method. The significance analysis compared with WT was performed using two-way ANOVA (** *p* < 0.01). Bars indicate standard error of the mean. The experiment was performed using three biological replicates.

To further understand whether *ZmCaM2-1* is involved in drought stress through the ABA-independent signaling pathway, the expression levels of ABA-inducible genes and drought-related genes were examined. As shown in Figure 9a–d, the relative expression levels of ABA-inducible genes (*ERD10*, *RAB18*, *COR47*, and *MYB*2) showed no observable differences between the *ZmCaM2-1*-overexpressing lines and the WT after exposure to 0.8 μm ABA at 0 h or 12 h. However, the drought-related gene *RD29A*, as an ABA-independent marker gene, was significantly down-regulated in the *ZmCaM2-1*-overexpressing lines under drought conditions at 24 h (Figure 9e). Taken together, overexpression of *ZmCaM2-1* can decrease the drought tolerance of *Arabidopsis* through the ABA-independent pathway.

## 3. Discussion

CaM and CMLs, as important Ca^2+^ sensors, play crucial roles in the response to abiotic stress [29]. Although many *CaM* and *CML* genes have been identified in various plants [30,31], the functions of those genes still need to be elucidated. Previously, we found that a *CaM* gene may be involved in drought stress tolerance in maize by using a transcriptome sequencing database [26,27]. However, its function remains unclear. In the present study, we cloned the *CaM* gene, namely *ZmCaM2-1* (Figure 1). The qRT-qPCR showed that the expression level of *ZmCaM2-1* is significantly up-regulated by drought treatment (Figure 2a). *ZmCaM2-1* was located in the cell nucleus and membrane, and can bind to Ca^2+^ (Figure 3 and Figure 4). *ZmCaM2-1*-overexpressing *Arabidopsis* shows reduced drought tolerance through increasing the ROS and MDA content, and decreasing the Pro content, POD activity, and SOD activity. (Figure 5, Figure 6 and Figure 7). Moreover, we found that *ZmCaM2-1*-overexpressing *Arabidopsis* shows normal sensitivity to ABA (Figure 8 and Figure 9). These findings reveal that *ZmCaM2-1* can negatively regulate the tolerance of maize to drought stress through the ABA-independent pathway.

Under stress conditions, the concentration of cytosolic Ca^2+^ rapidly increases and is recognized by CaM and CMLs. Ca^2+^/CaM activates the target genes and initiates a series of physiological responses [32,33,34]. Extensive studies have shown that the activation of CaM/CMLs depends on the binding properties of Ca^2+^. Hsp70 was reported to bind to AtCaM2 in a Ca^2+^-dependent manner [35]. MYB2 was also found to interact with CaM in the presence of Ca^2+^ [36,37]. In addition, the subcellular location of CaM/CMLs also can affect Ca^2+^ binding and signaling transduction. CaM/CMLs have been found to be located in the cytoplasm, cell membrane, and nucleus [38]. For example, MpCML40 was located in the plasma membrane and in the nucleus [39]. CaCML13 was found to be located in the plasma membrane, cytoplasm, and nucleus [40]. In this study, we found that ZmCaM2-1 is located in the cell nucleus and membrane and can bind to Ca^2+^ (Figure 3 and Figure 4). The subcellular localization of ZmCaM2-1 may be beneficial for binding to Ca^2+^, and the Ca^2+^/ZmCaM2-1 complex may activate the Ca^2+^ signal transduction pathway to respond to drought stress in maize.

An increasing number of studies have shown that *CaM/CMLs* positively regulate the drought stress response. Overexpression of *EcCaM* can enhance the tolerance of *Arabidopsis* to drought and salt stresses [41]. *OsCML16* can positively regulate the drought stress tolerance in rice [42]. However, several *CaM/CMLs* were found to act as negative regulators of drought tolerance. For example, *GsCML27*-overexpressing *Arabidopsis* shows increased sensitivity to osmotic stress [43]. *CML20* negatively regulates drought stress tolerance in *Arabidopsis* [44]. Thus, *CaM/CMLs* may have different functions in the response to drought stress. In this study, *ZmCaM2-1*-overexpressing *Arabidopsis* shows decreased drought stress tolerance (Figure 5 and Figure 6), indicating that *ZmCaM2-1* has a negative effect on the tolerance to drought stress. The result will provide a reference for us to understand the function of *ZmCaM2-1* in maize.

ROS as a second messenger is important to protect plants from various abiotic stresses [45,46]. However, the excessive accumulation of ROS can cause cell damage [47]. Overexpression of *CIPK11* can confer reduced drought tolerance by enhancing ROS accumulation [48]. Overexpression of *MePP2C24* enhances the sensitivity of *Arabidopsis* to drought stress with a higher ROS content [49]. *VvWRKY18*-overexpressing *Arabidopsis* exhibited a decreased tolerance to drought stress and an increased level of ROS [50]. In this study, we found that overexpression of *ZmCaM2-1* decreases the tolerance of *Arabidopsis* to drought stress with a higher MDA content, lower Pro content, lower POD and SOD activity, and greater ROS production. These results demonstrate that *ZmCaM2-1* negatively regulates the tolerance to drought stress through increasing the ROS accumulation. It is well known that ROS can increase the concentration of Ca^2+^, and Ca^2+^ also can trigger ROS generation [51,52]. Both Ca^2+^ and ROS are involved in drought stress in plants [53]. Thus, *ZmCaM2-1* may regulate the Ca^2+^ and ROS signaling pathway to participate in the drought stress response.

ABA plays a crucial role in the response to various abiotic stresses [54]. Both ABA-dependent and ABA-independent signaling pathways are utilized in the response to osmotic stress [55]. Our data have indicated that the expression of *ZmCaM2-1* is not significantly induced by ABA treatment (Figure 2c). *ZmCaM2-1*-overexpressing transgenic *Arabidopsis* shows normal sensitivity to ABA treatment (Figure 8). Under ABA treatment, there was no significant difference in the transcription level of ABA-responsive genes between the *ZmCaM2-1*-overexpressing lines and the WT (Figure 9a–d). These results indicate that *ZmCaM2-1* may negatively regulate drought stress tolerance in an ABA-independent manner. The results are consistent with the function of *OsCML4* [22]. Future research should focus on how *ZmCaM2-1* modulates drought stress in an ABA-independent manner.

## 4. Materials and Methods

### 4.1. Plant Materials and Stress Treatments

Seeds of the maize inbred line B73 and the wild-type (WT) *Arabidopsis* (Columbia) were provided by the Maize Breeding Innovation Team of Jilin Agricultural University. The maize inbred line B73 was planted in a germination box with a light/dark cycle of 16/8 h at 28/25 °C. The seedlings of B73 at the third-leaf stage (V3) were treated by PEG6000 (20% *w*/*v*) [56], ABA (50 μM) [56], and NaCl (250 mM) [56] and by low-temperature stress (4 °C) [57]. The leaves with the same growth tendency were taken at 0 h, 6 h, 12 h, 24 h, and 48 h, and stored at −80 °C.

### 4.2. RNA Extraction and qRT-PCR

Total RNA was extracted from the plant leaves using Trizol (Tiangen, Beijing, China), and 2 μg RNA was reverse-transcribed into cDNA using a reverse transcription kit (TOYOBO, Shanghai, China). The qRT-PCR was carried out using QuantStudio 3 (Thermo, Waltham, MA, USA). The data were calculated using the 2^−ΔΔCT^ method, based on three biological replicates [58]. *ZmTUB* (GRMZM2G066191) and *ACTIN2* (At3g18780) were used as internal controls. All primer sequences (*ZmTUB*-F/R, *ACTIN2*-F/R, and *ZmCaM2-1*-Q-F/R) are shown in Table S1.

### 4.3. ZmCaM2-1 Cloning and Bioinformatics Analysis

The full-length sequence of *ZmCaM2-1* was cloned from the maize inbred line B73 via reverse transcription–polymerase chain reaction (RT-PCR). All primer sequences are shown in Table S1 (*ZmCaM2-1*-Cloning-F/R). The amino acid sequence of ZmCaM2-1 was analyzed using Uniprot (https://www.uniprot.org/, accessed on 11 November 2024). The ZmCaM2-1 homologous sequences from other plants were searched using NCBI-BLAST (https://www.ncbi.nlm.nih.gov/, accessed on 11 November 2024), and a phylogenetic tree was constructed using MEGA 11. Sequence alignments of ZmCaM2-1 orthologs were also performed using MEGA 11 software.

### 4.4. Purification of ZmCaM2-1 Protein and Ca^2+^ Binding Assay

To detect whether the ZmCaM2-1 protein can bind to Ca^2+^, the CDS of *ZmCaM2-1* was constructed into the restriction sites (*Nde* I and *BamH* I) of the pET29b vector using a Seamless Cloning Kit (Beyotime, Shanghai, China). All primer sequences (pET-29b-*ZmCaM2-1*-F/R) are shown in Table S1. The recombinant vector pET29b-ZmCaM2-1 was transformed into the BL21 (*DE3*) competent cell to generate the ZmCaM2-1-His protein (Coolaber, Beijing, China). The recombinant protein ZmCaM2-1-His was induced with 1 mM Isopropyl-beta-D-thiogalactopyranoside for 4 h at 37 °C, and the protein was purified using a His-Tag protein Purification Kit (LABLEAD, Beijing, China). The purified ZmCaM2-1-His protein (2 μg) was separated using 15% SDS-PAGE, which was added to either CaCl_2_ (1 mM, 10 mM, or 40 mM) or 3 mM EGTA. In addition, the purified ZmCaM2-1-His protein (1 μg, 2 μg, and 4 μg) was separated in the 15% SDS-PAGE, which was added to 1 mM CaCl_2_ or 3 mM EGTA.

### 4.5. Subcellular Localization of ZmCaM2-1

For the subcellular localization assay, the CDS of *ZmCaM2-1* was constructed into the restriction sites (*Bgl* II and *Spe* I) of the pCAMBIA1302 vector to generate the ZmCaM2-1-GFP protein using a Seamless Cloning Kit (Beyotime, Shanghai, China). The primers are listed in Table S1 (pCAMBIA1302-*ZmCaM2-1*-F/R). The plasmids were transformed into the leaves of a 4-week-old *Nicotiana benthamiana* using an *Agrobacterium*-mediated method [59]. The leaves were cultured in darkness at 22 °C for 16–24 h. In addition, the CDS of *ZmCaM2-1* was constructed into the restriction sites (*Pst* I and *Bam*H I) of the 16318-hGFP vector to generate the 35S:: ZmCaM2-1-GFP protein using a Seamless Cloning Kit (Beyotime, China). The primers (35S:: ZmCaM2-1-GFP-F/R) and sequences are listed in Table S1 and Figure S2. The plasmids were transformed into *Arabidopsis* protoplasts according to the method described by Yoo et al. (2007) [60]. The GFP signal was detected and imaged using a confocal laser scanning microscope (Leica, Frankfurt, Germany).

### 4.6. Gene Transformation and Drought Tolerance Assay

To generate transgenic *Arabidopsis*, the CDS of *ZmCaM2-1* was constructed into the restriction sites (*Nco* I and *Pml* I) of the pCAMBIA3301-4Myc vector using a Seamless Cloning Kit (Beyotime, China). The primers (pCAMBIA3301-4Myc-*ZmCaM2-1*-F/R) and sequences are listed in Table S1 and Figure S2. The recombinant plasmid was transferred into the *EHA105* competent cell (Coolaber, China) and then transformed into the wild-type (WT) *Arabidopsis* (Columbia) using the floral dip method [61]. T3-generation transgenic *Arabidopsis* lines were obtained through screening and self-crossing, which were verified using phosphine oxalate, a Bar test strip, and qRT-PCR (*ZmCaM2-1*-Q-F/R).

The sterile WT and T3-generation transgenic *Arabidopsis* seeds OE1 and OE2 were germinated in 1/2 Murashige and Skoog (MS) medium (pH 5.7) with 200 mM and 300 mM mannitol or 0.5 μM and 0.8 μM ABA treatments. The root length and leaf expansion rate were measured after treatment for 10 d. For a drought tolerance assay, 7-day-old seedlings grown on agar plates were transferred to soil and placed in a growth chamber with a 16-hour/8-hour light/dark cycle at 22 °C for 2 weeks. The 3-week-old plants were subjected to drought treatment by withholding water for 14 d, followed by re-watering for 3 d. Images were captured using a Nikon D7000 (Nikon, Tokyo, Japan).

### 4.7. Physiological Index Detection

About 0.1 g of plant leaves was used to determine the SOD, POD, MDA, Pro, and ROS activity. The Pro content was measured using the method of L. S. Bates et al. (1973) [62]. Pro can react with ninhydrin to form red compounds, and the absorption value is measured at 520 nm. The MDA content was determined using thiobarbituric acid (TBA). MDA can react with TBA to produce reddish-brown 3,5,5-Trimethyloxazolidine-2,4-dione (Trimethadione). The maximum absorption value was measured at 532 nm and corrected at 600 nm and 450 nm [63]. The SOD activity was measured via inhibiting reduction of nitrogen blue tetrazole (NBT) under light. The absorbance value was measured at 560 nm [64]. The POD activity was measured using the guaiacol method. In the presence of POD, H_2_O_2_ can oxidize o-methoxy-phenol (guaiacol) to produce reddish-brown 4-o-methoxyphenol. The absorption value was measured at 470 nm [65]. ROS were extracted using a Plant ROS ELISA Kit according to the manufacturer’s instructions (KETE, Wenzhou, China). The ROS content was detected at 450 nm using a full-wavelength enzyme-labeling apparatus (HBS-ScanY, Shanghai, China). All samples were calculated using three biological replicates.

### 4.8. Statistical Analysis

The statistical experiments were analyzed according to three biological replicates. All data were analyzed using GraphPad Prism 9.0 software with one- and two-way ANOVAs. A significant difference was defined as * *p* < 0.05 and a highly significant difference as ** *p* <  0.01.

## 5. Conclusions

Our results showed that *ZmCaM2-1* negatively regulates drought tolerance by reducing antioxidant enzyme activity and increasing ROS content. Moreover, *ZmCaM2-1* is involved in the drought stress response in an ABA-independent manner. Future studies will involve the function of *ZmCaM2-1* in the drought stress response in maize. *ZmCaM2-1* may be beneficial for breeding drought-tolerant maize varieties through gene editing technology. This finding will provide a piece of information for understanding the Ca^2+^ and ABA-independent signaling pathways involved in plant stress responses and is helpful for studying the function of CaMs in plants.

## Data Availability

Data are contained within the article or Supplementary Materials.

## Supplementary Materials

The supporting information can be downloaded at: https://www.mdpi.com/article/10.3390/ijms26052156/s1.

## Abbreviations

The following abbreviations are used in this manuscript:

Ca^2+^	Calcium ion
CaM	Calmodulin
CML	Calmodulin-like protein
CBL	Calcineurin B-Like
CDPK	Calcium-dependent protein kinase
ABA	Abscisic acid
ROS	Reactive oxygen species
CDS	Coding sequence
qRT-PCR	Quantitative Real-Time PCR
GFP	Green fluorescent protein
h	Hour
EGTA	Ethylene glycol tetraacetic acid
WT	*Arabidopsis thaliana* (Columbia, Col-0)
OE	*ZmCaM2-1*-Overexpressing *Arabidopsis*
d	Day
TBA	Thiobarbituric acid
Pro	Proline
MDA	Malondialdehyde
SOD	Superoxide dismutase
POD	Peroxidase
