# Supplementary material for: ZmCaM2-1, a Calmodulin Gene, Negatively Regulates Drought Tolerance in Transgenic Arabidopsis Through the ABA-Independent Pathway
Source: Int J Mol Sci. 2025 Feb 27;26(5):2156. doi: 10.3390/ijms26052156 (PMC11900298; doi:10.3390/ijms26052156)
Supplement: Supplementary file 1 [file ijms-26-02156-s001.zip › Supplementary Figure S1.pdf]

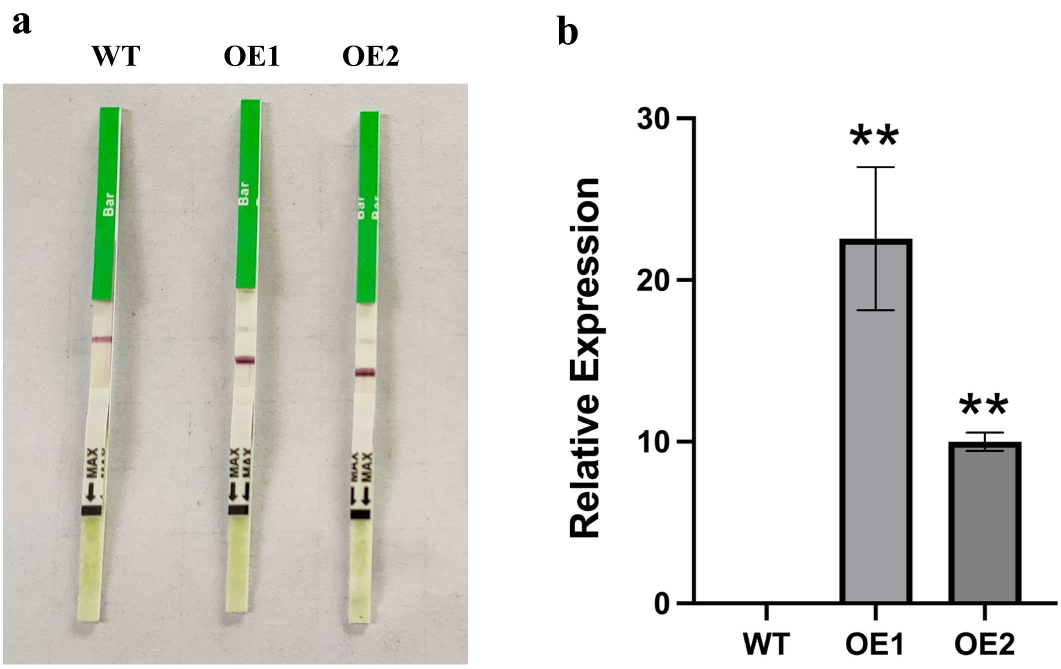

Figure. S1 Detection of T3 generation transgenic *Arabidopsis*. (a) Dipstick strips were used to detect transgenes. (b) Relative expression levels of *ZmCaM2-1* in the leaf of the transgenic lines OE1 and OE2. The analysis of significance compared with WT was performed using one-way ANOVA (** $p < 0.01$). Bars indicate the standard deviation of the mean. The experiment was performed using three biological replicates.
