# Supplementary material for: ZmCaM2-1, a Calmodulin Gene, Negatively Regulates Drought Tolerance in Transgenic Arabidopsis Through the ABA-Independent Pathway
Source: Int J Mol Sci. 2025 Feb 27;26(5):2156. doi: 10.3390/ijms26052156 (PMC11900298; doi:10.3390/ijms26052156)
Supplement: Supplementary file 1 [file ijms-26-02156-s001.zip › Supplementary Figure S2.pdf]

a

ACAGCCCAAGCTTGCATGCCTGCAGATGGCGGACCAGCTCACCGACGAACAGATCGCCGAGTTCAAGG
AGGCCTTCAGCCTCTTCGACAAGGACGGCGACGGCTGCATCACCACCAAGGAACTTGGCACTGTGATG
CGCTCATTGGGGCAGAACCCTACTGAGGCTGAGCTTCAGGACATGATCAATGAGGTTGATGCTGATGGC
AATGGAACCATCGACTTTCCTGAGTTTCTCAACCTGATGGCACGCAAGATGAAGGACACCGACTCTGA
GGAGGAGCTCAAGGAGGCCTTCCGTGTGTTCGACAAGGACCAGAACGGCTTCATCTCCGCTGCTGAGC
TTCGCCATGTCATGACCAACCTAGGCGAGAAGCTGACGGACGAGGAGGTGGACGAGATGATCCGTGAA
GCCGACGTGGACGGTGATGGCCAGATCAACTACGACGAGTTCGTGAAGGTGATGATGGCCAAGGGATC
CATGGTGAGCAAGGGCGAGG

b

CGGGGGACTCTTGACCATGGCAATGGCGGACCAGCTCACCGACGAACAGATCGCCGAGTTCAAGGAG
GCCTTCAGCCTCTTCGACAAGGACGGCGACGGCTGCATCACCACCAAGGAACTTGGCACTGTGATGCG
CTCATTGGGGCAGAACCCTACTGAGGCTGAGCTTCAGGACATGATCAATGAGGTTGATGCTGATGGCAA
TGGAACCATCGACTTTCCTGAGTTTCTCAACCTGATGGCACGCAAGATGAAGGACACCGACTCTGAGG
AGGAGCTCAAGGAGGCCTTCCGTGTGTTCGACAAGGACCAGAACGGCTTCATCTCCGCTGCTGAGCTT
CGCCATGTCATGACCAACCTAGGCGAGAAGCTGACGGACGAGGAGGTGGACGAGATGATCCGTGAAG
CCGACGTGGACGGTGATGGCCAGATCAACTACGACGAGTTCGTGAAGGTGATGATGGCCAAGTGCCAC
GTGGGGTTAATTAACGGTGAACAAAAGCTAATCTCCGAGGAAGACTTGAACGGTGAACAAAAATTAAT
CTCAGAAGAAGACTTGAACGGACTCGACGGTGAACAAAAGTTGATTTCTGAAGAAGATTTGAACGGT
GAACAAAAGCTAATCTCCGAGGAAGACTTGAACGGTAGCGCTTAG

Figure. S2 The *ZmCaM2-1* sequence was successfully constructed into 16318-hGFP vector and pCAMBIA3301-4Myc vector, respectively. (a) Sequencing result of recombinant vector 35S:: ZmCaM2-1-GFP. (b) Sequencing result of recombinant vector pCAMBIA3301-4Myc-ZmCaM2-1. The red fonts are the expanded area.
